# Parental care experience of children with type 1 diabetes: a qualitative meta-synthesis

**DOI:** 10.1590/1980-220X-REEUSP-2024-0118en

**Published:** 2024-11-11

**Authors:** Zhaoying Zhang, Xin Wang, Wenwen Dong, Danshan Gao

**Affiliations:** 1 Zhengzhou University, School of Nursing and Health, Zhengzhou, China.

**Keywords:** Diabetes Mellitus, Type 1, Child, Parents, Emotions, Qualitative Research, Review, Diabetes Mellitus Tipo 1, Criança, Pais, Emoções, Pesquisa Qualitativa, Revisão, Diabetes Mellitus Tipo 1, Niño, Padres, Emociones, Investigación Cualitativa, Revisión

## Abstract

**Objective:**

To assess qualitative studies on parents’ caregiving experiences whose children have T1DM and develop personalized support strategies based on the findings.

**Method:**

A systematic review with meta-synthesis performed in the Cochrane Library, Embase, Scopus, CINAHL, PubMed, Web of Science, CNKI, CBM, VIP, and Wanfang databases. Quality was assessed via the JBI criteria, and meta-aggregative method was applied to categorize the results into subtopics and aggregate into three interrelated meta-topics to understand parents’ caregiving experiences.

**Results:**

In total, 2,100 articles were found, out of which 15 were selected and analyzed. The identified three meta-topics were “Parents facing multiple physical, mental and life challenges”, “Parents’ lack of a full range of external support” and “Parents’ caregiving role competency enhanced to adjust to the new life”.

**Conclusion:**

it is critical for healthcare professionals to recognize these parental experiences and offer targeted knowledge, skills training, and psychological support tailored to their needs, including group training, online mindfulness interventions, and improved empathy from the medical team.

## RESUMO

**Objetivo:**

Avaliar estudos qualitativos sobre experiências de cuidado de pais cujos filhos têm DM1 e desenvolver estratégias de suporte personalizadas com base nos achados.

**Método:**

Revisão sistemática com metassíntese realizada nas bases de dados Cochrane Library, Embase, Scopus, CINAHL, PubMed, Web of Science, CNKI, CBM, VIP e Wanfang. A qualidade foi avaliada por meio dos critérios do JBI, e o método meta-agregativo foi aplicado para categorizar os resultados em subtópicos e agregar em três metatópicos inter-relacionados para compreender as experiências de cuidado dos pais.

**Resultados:**

No total, foram encontrados 2.100 artigos, dos quais 15 foram selecionados e analisados. Os três metatópicos identificados foram “Pais enfrentando múltiplos desafios físicos, mentais e de vida”, “Falta de uma gama completa de suporte externo pelos pais” e “A competência do papel de cuidador dos pais aprimorada para se ajustar à nova vida”.

**Conclusão:**

É fundamental que os profissionais de saúde reconheçam essas experiências parentais e ofereçam conhecimento específico, treinamento de habilidades e apoio psicológico adaptado às suas necessidades, incluindo treinamento em grupo, intervenções de atenção plena online e maior empatia da equipe médica.

## RESUMEN

**Objetivo:**

Evaluar estudios cualitativos sobre las experiencias de cuidado de padres cuyos hijos tienen diabetes tipo 1 y desarrollar estrategias de apoyo personalizadas basadas en los hallazgos.

**Método:**

Revisión sistemática con metasíntesis realizada en las bases de datos Cochrane Library, Embase, Scopus, CINAHL, PubMed, Web of Science, CNKI, CBM, VIP y Wanfang. La calidad se evaluó utilizando los criterios del JBI y se aplicó el método metaagregativo para categorizar los resultados en subtemas y agregarlos en tres metatemas interrelacionados para comprender las experiencias de cuidado de los padres.

**Resultados:**

En total se encontraron 2.100 artículos, de los cuales 15 fueron seleccionados y analizados. Los tres metatemas identificados fueron “Padres que enfrentan múltiples desafíos físicos, mentales y vitales”, “Falta de una gama completa de apoyo externo por parte de los padres” y “Competencia de los padres en la función de cuidado mejorada para adaptarse a la nueva vida”.

**Conclusión:**

Es fundamental que los profesionales de la salud reconozcan estas experiencias de los padres y ofrezcan conocimientos específicos, capacitación en habilidades y apoyo psicológico adaptado a sus necesidades, incluida capacitación en grupo, intervenciones de atención plena en línea y una mayor empatía por parte del personal médico.

## INTRODUCTION

Type 1 Diabetes Mellitus (T1DM) indeed poses significant challenges not only to the children affected by it but also to their families, especially parents, who play a crucial role in managing the disease. T1DM is the predominant endocrine disorder in the pediatric population, characterized by its chronic^(1)^, lifelong, and autoimmune nature^(2)^. It affects approximately 8.4 million individuals globally, of which 1.5 million are children^(3)^. The chronic and autoimmune nature of T1DM demands constant vigilance over blood glucose levels, dietary management, and insulin administration, which are further complicated in the context of a child’s developing physical and psychological state^(4,5,6)^.The responsibilities of parents caring for children with T1DM are multifaceted, encompassing the adjustment of insulin levels in response to blood glucose concentrations, dietary and physical activity regulation, complication prevention, and emotional support provision^(7)^. Prolonged exposure to the demands of caregiving can precipitate a spectrum of adverse experiences among parents, including denial, avoidance, anxiety, depression, and sustained stress^(8,9)^, often leading to maladaptive coping mechanisms^(10)^. These persistent negative experiences and coping strategies not only undermine the parental capacity to nurture children with resilience but also significantly impair disease management quality, thereby detracting from continuous and high-quality care provision. Moreover, T1DM’s impact extends beyond individuals and affects family dynamics, often altering family routines and having broader implications for family life.

Currently, research focusing on parents of children with T1DM predominantly centers on quantitative analyses of their quality of life^(11)^ and psychological well-being^(12)^. However, qualitative investigations into the nuanced caregiving experiences and the complexities of family life remain underexplored. Consequently, there exists a notable gap in actionable insights into the psychological framework, behavioral patterns, and motivational drives of these parents, which are crucial for developing precise and effective interventions. Addressing this gap is imperative for enabling parents to swiftly adapt to their caregiving roles, thereby optimizing health outcomes for children with T1DM.

Therefore, gaining an in-depth comprehension of the caregiving experiences among parents of children with T1DM is essential. It adds a critical layer of understanding to their parenting dynamics, paving the way for creating more efficacious T1DM management strategies and interventions. However, given the limitations of a single qualitative study in fully capturing the complexities and difficulties associated with parental care, a meta-analysis was conducted to systematically review the literature on qualitative studies exploring the experiences of parents caring for children with T1DM by synthesizing findings from various qualitative studies within this domain, aiming to assess qualitative studies on parents’ caregiving experiences whose children have T1DM and develop personalized support strategies based on the findings.

## METHOD

### Study Design

In this systematic review, the meta-aggregative method was adopted. In the cluster process, the findings were aggregated into subtopics by descriptive and conceptual similarity, and then grouped into broader and more comprehensive topics. When looking for similarities and differences between the perspectives of the different social actors who experienced the phenomenon under investigation, subtopics and topics were integrated instead of compared. For its development, the following stages were considered: 1) Approach to the topic and research question formulation (What are the physiological and psychological reactions and challenges that parents face during the whole process of caring for a child with T1DM?); 2) Definition of information assessed in the articles; 3) Selection of databases and descriptors; 4) Selection of studies based on previously established criteria; 5) Assessment of the methodological rigor of empirical studies; 6) Collection and organization of information in relevant articles; 7) Synthesis of findings with development of qualitative meta-synthesis. Although the project of this review was not registered on the PROSPERO platform, all stages proposed by this entity were followed, and are best described in the following sections.

### Article Inclusion and Exclusion Criteria

Inclusion criteria are as follows: 1) Population – healthy parents of children with T1DM (age < 18 years); 2) Phenomenon of Interest – experiences of parents of children with T1DM on life care, psychological coping, and challenges; 3) Context – children with T1DM are taken care of by their parents; 4) Outcome – subjective description of the perception of phenomenon; 5) Research types – phenomenological research, grounded theory, case study, ethnography, action research and other qualitative research methods.

Exclusion criteria are as follows: 1) There are only abstracts but no full texts; 2) Literature with repeated publications or incomplete data; 3) Non-Chinese and English literature.

### Search Strategies

Initially, the search was performed in January 2024. The assessment and analysis of results were performed between February and April 2024. The following combination of descriptors (MeSH) in English was used to conduct the search in PubMed and in other databases with small adaptations, according to their specificities, such as “Diabetes Mellitus” AND “Child” AND “Parents” AND “Experience” AND “Qualitative Research”, with date limits [from database building to 31/1/2024], species [humans] and language [English or Chinese]. Ten databases were used, such as Cochrane Library, Embase, Scopus, CINAHL, PubMed, Web of Science, CNKI, CBM, VIP and Wang Fang. Taking PubMed as an example, the specific search strategies are shown in Chart 1.

**Chart 1:** Retrieval strategy of PubMed.

Procedure	Search mode
#1	Diabetes Mellitus, Type 1 [mh] OR Diabetes, Type 1 [tiab] OR Type 1 Diabetes [tiab] OR Type 1 Diabetes Mellitus [tiab] OR Diabetes Mellitus, Type I [tiab] OR Juvenile Onset Diabetes [tiab] OR Diabetes Mellitus, Juvenile Onset [tiab] OR Insulin-Dependent Diabetes Mellitus [tiab] OR Insulin Dependent Diabetes Mellitus 1 [tiab] OR Diabetes Mellitus, Insulin Dependent [tiab]
#2	child [mh] OR Children [tiab] OR Infant [tiab] OR Infants [tiab] OR Adolescent [tiab] OR Teenager [tiab]
#3	parents [mh] OR parent [tiab] OR father [tiab] OR mother [tiab] OR maternal* [tiab] OR paternal* [tiab]
#4	Emotions [tiab] OR experience [tiab] OR needs [tiab] OR perceptions [tiab] OR feeling [tiab]
#5	qualitative research [mh] OR qualitative study [tiab] OR research, qualitative [tiab] OR phenomenon* [tiab] OR ground theory [tiab] OR narrative research [tiab] OR action research [tiab] OR case study [tiab] OR descriptive study [tiab] OR focus group [tiab] OR interview[tiab]
#6	#1 AND #2 AND #3 AND #4 AND #5

### Article Selection and Quality Assessment

Literature screening, data extraction and cross-checking were conducted independently by two researchers trained in evidence-based methodology. In case of disagreement, a third researcher was consulted to assist in judgment. In the literature selection process, the title should be read first, and the abstract and full text should be read later, after excluding obviously irrelevant literature to determine whether the final inclusion is included.

The included literature was independently assessed by two researchers trained in evidence-based practice methodology according to the 2016 edition of the Australian JBI Centre for Qualitative Research Quality Evaluation Criteria for Evidence-Based Health Care. In case of conflicting assessment results, a third researcher decides. Each item is assessed as “yes”, “no”, “unclear”, “not applicable”. The above criteria are fully met, and the possibility of various biases is as low as grade A; partially satisfied and the possibility of bias were grade B; those who are completely unsatisfied and have a high probability of bias are classified as grade C. Grade C studies were eliminated (Chart 2).

**Chart 2:** Methodological quality assessment of included literature(n = 15).

Inclusion study	①	②	③	④	⑤	⑥	⑦	⑧	⑨	⑩	Literature quality
Sullivan-Bolya et al.^(13)^	Yes	Yes	Yes	Yes	Yes	No	No	Yes	Yes	Yes	B
Khandan et al.^(14)^	Yes	Yes	Yes	Yes	Yes	No	No	Yes	Yes	Yes	B
Tong et al.^(15)^	Yes	Yes	Yes	Yes	Yes	No	No	Yes	No	Yes	B
Iversen et al.^(16)^	Yes	Yes	Yes	Yes	Yes	No	No	Yes	Yes	Yes	B
Rifshana et al.^(17)^	Yes	Yes	Yes	Yes	Yes	No	No	Yes	Yes	Yes	B
Symons et al.^(18)^	Yes	Yes	Yes	Yes	Yes	No	No	Yes	Yes	Yes	B
Nurmi and Stieber-Roger^(19)^	Yes	Yes	Yes	Yes	Yes	No	No	Yes	Yes	Yes	B
Smaldone and Ritholz^(20)^	Yes	Yes	Yes	Yes	Yes	No	No	Yes	Yes	Yes	B
Moghadam et al.^(21)^	Yes	Yes	Yes	Yes	Yes	No	No	Yes	Yes	Yes	B
Tong et al.^(22)^	Yes	Yes	Yes	Yes	Yes	No	No	Yes	Yes	Yes	B
Rossiter et al.^(23)^	Yes	Yes	Yes	Yes	Yes	Yes	No	Yes	Yes	Yes	B
Sullivan-Bolyai et al.^(24)^	Yes	Yes	Yes	Yes	Yes	No	No	Yes	Yes	Yes	B
Chan et al.^(25)^	Yes	Yes	Yes	Yes	Yes	No	No	Yes	Yes	Yes	B
Dai et al.^(26)^	Yes	Yes	Yes	Yes	Yes	No	No	Yes	No	Yes	B
Haegele et al.^(27)^	Yes	Yes	Yes	Yes	Yes	No	No	Yes	Yes	Yes	B

Caption: ① Whether the philosophical basis is consistent with the methodology; ② Whether the methodology is consistent with the research question or research objective; ③ Whether the methodology is consistent with the data collection method; ④ Whether the methodology is consistent with data representativeness and typicality and data analysis methods; ⑤ Whether the methodology is consistent with the interpretation of results; ⑥ Whether to explain the researcher’s own situation from the perspective of cultural background and values; ⑦ Whether the impact of the researcher on the research or the impact of the research on the researcher is stated; ⑧ Whether the research object and its views are typical; ⑨ Whether it is approved by the ethics committee; ⑩ Whether the conclusion is derived from data analysis and interpretation.

### Data Analysis and Processing

Two reviewers read and reviewed the articles independently for systematic data extraction and recording. In case of disagreement, a third researcher was consulted to assist in judgment. Data extraction mainly includes author, country, research method, research object, interest phenomenon, interview place, and main results.

Finally, the following three meta-topics were identified: “Parents facing multiple physical, mental and life challenges”; “Parents’ lack of a full range of external support”; and “Parents’ caregiving role competency enhanced to adjust to the new life” (Chart 3).

**Chart 3:** Process of primary data cluster.

Findings	Subtopic	Topics	Meta-topic
There are times when I’m completely drained. I sit down and cry. I ask God, “Why me?”. It’s challenging (M06-mother of a 5-year-old girl)^(25)^.	Sensing body fatigue	At-risk physical health	Parents facing multiple physical, mental and life challenges
I have become an insomniac. I don’t sleep at night as every 2–3 hours I have to check on him (…) I sleep only 2–3 hours. It’s my son’s diabetes that has made me like that (M07-mother of a 9-year-old boy)^(25)^.	Chronic sleep deprivation		
I could not believe that my child had diabetes. I took him to different doctors (One mother)^(21)^.	Diagnosis difficult to accept, deny and escape	Complex emotional experience	
It was like you were killing your own daughter…. I don’t think that I’m going to be able to do it (One mother)^(20)^.	Fear of managing the disease process		
Society’s perception of diabetes is backward and even wrong. Her peers were afraid to drink from her glass as they thought that it was contagious… (M10-mother of a 10-year-old girl)^(14)^.	The outside world’s misunderstanding of the disease		
I was so worried about the consequences of spreading the news among them, which may have a negative or bad effect on my daughter’s future (FATIMA)^(23)^.	Concerns about stigma associated with type 1 Diabetes Mellitus		
I feel guilty that I may have done something wrong that has resulted in my child’s diabetes as I’m the one who feeds him and takes care of him (M07-mother of a 9-year-old boy)^(25)^.	Self-blame and guilt		
I went through a grieving process, not grief for dying... it’s about the loss of your child’s health (One mother)^(18)^.	Sorrow and pity		
Diabetes is an underrated condition: you look healthy—nothing is visible. To be parents in this situation implies that you live in a state of perpetual attention (Mother child 7)^(13)^.	Constant high tension		
There are family and friends ready when we need time for ourselves. But it is difficult to let go, and we think about all the considerations we must make all the time. It is difficult for us to leave this responsibility to others (Mother child 7)^(16)^.	Alleviating the burden of care and the ambivalence of not being able to let go		
I am always thinking about his future. I wonder what will happen to his body. Can he be successful in his life? I do not know; the future is unclear (M:3)^(14)^.	Fear for the child’s future		
We are confused about why blood sugar control is not very good. I don’t know what to do, I want to know all about blood sugar control (One mother)^(26)^.	Lack of knowledge about the disease	Taking care of lack of knowledge and ability	
I was confused at the beginning and did not know where to start. I had never practiced insulin injection before. I still remember how my hands trembled when I injected my child the first time, causing me to inject too much insulin (DM17)^(15)^.	Care skills need to be improved		
Now I rarely focus my energy on the company [I work for]; just get off work as soon as possible. I used to leave early and return late to make money. Now I quit my job and found a new one that can make a living while taking care of my child (DM5)^(21)^.	Changing future career plans	Limited personal development and social activities	
Since my child had a ketosis coma, I have never dared to relax again. I have lost myself. Every day in the year, there are no more visits to relatives or friends (DM10)^(22)^.	Interfering with normal social interaction		
I revolve around my children almost all day, and I don’t have any time to myself (One mother)^(18)^.	Reducing personal time elasticity		
I spend a lot of time with Ahmed. This has made his siblings dissatisfied (One mother)^(21)^.	Having an impact on siblings	The reconstruction of family dynamics	
We ordered beepers and we would beep. Every time…(she) did a (blood glucose) test we would beep the number… she’d just beep the number and I would call her and we would consult each other (A father of a 4-year-old child)^(20)^.	Spousal responsibility division and support		
I have too many chores and things to take care of besides taking care of him, but it is difficult for me to juggle them (M07-mother of a 9-year-old boy)^(25)^.	Hard to balance care and daily life		
What shall I say… near fights where we have accused each other like. Why did you forget this? Don’t you know that this food contains lots of carbohydrates? Yes, we have had a few of those (Father child 8)^(16)^.	Diabetes-related family conflict		
The test strip and the insulin needle are too expensive. We did not have such costs earlier, but now we have to save by cutting down on family expenses for the sake of our child (M:7)^(14)^.	Financially stressful long-term treatment	Heavy economic burden	
… a lot of things I don’t think he (father) does right with this, and I’m sure he thinks I micro-organize the whole thing… he doesn’t get the calls from the nurse. He’s not watching the meter all day with every number that’s coming up, and so I think it’s a little more stress free for him (One mother of a 9-year-old girl)^(20)^.	Absent paternal care role	Insufficient family support	Parents’ lack of a full range of outside support
My son is a little uncomfortable; they (family) are putting the responsibility on me, the child does not cooperate with me, I can do nothing, they should understand my difficulties more (One mother)^(26)^.	Relatives’ lack of understand		
I hope the whole family can help me share a little, because my ability is limited, and if my family members can help take care of me, I can feel more relaxed and the care effect will be better (One mother)^(26)^.	Family members’ hope to participate in care		
I really want the doctors and nurses to tell me what kind of food that my child can and cannot eat, in what amount, and how to prepare it, so I can write them all down in a little notebook. I have a hard time figuring out the calories of the diet. I wish I could ask the doctors and nurses to prepare a list of recipes so I can follow them; otherwise, it is too difficult for me to prepare the meals for my child (DM7)^(15)^.	Eager for professional guidance from medical staff	Insufficient professional support	
Nowadays, we can find all kinds of information on the Internet, but there are always examples of falsehoods on the Internet. The more the information that I browse, the more I want to know, and I feel panicky deep down. I particularly hope that there will be a professional platform where I can ask question when I encounter problems that I cannot handle and receive timely answers from doctors and nurses (DM8)^(15)^.	Difficult to obtain authoritative information		
The children do not have medical insurance; these test strips, insulin cannot be reimbursed. It would be nice if some drugs were covered by Medicare, as they are for type 2 diabetes (One mother)^(26)^.	Lack of social security	Insufficient social support	
Since my child has started going to school, my heart really trembles as soon as the school calls, and I am afraid that my child will have an accident. Teachers at school do not know how to take care of diabetic children, and the schools generally have no nurse specialist. I am really afraid that there will be an accident (DM13)^(15)^.	Not perfect school configuration		
The family will tell me that you are the mother of the child and you must take care of him (One mother)^(17)^.	Relatives taking their parents’ care for granted	Unreasonable external expectations	
I feel anxious every time I go to the clinic. They (healthcare staff ) don’t understand the reality of living with a T1DM child, that I’ve done my best and still don’t meet the requirements, and he feels that I should be able to take care of the child (One mother)^(20)^.	Unrealistic expectations form medical staff		
But over the time, the situation has been changed and become almost ordinary and acceptable to us (KHULUD)^(23)^.	Gradual acceptance of disease	Self-empowerment, actively responding to life changes	Parents’ caregiving role competency enhanced to adjust to the new life
Taking care of my daughter, it’s not that I think I can or can’t, but that I must be able to do. It’s like a parent’s obligation. If I can’t, what else can she do? It’s not something I can or can’t do. It’s something I must be able to do (DM6)^(22)^.	Adapt to the caregiving role		
As time went by, I felt that I could handle the problems in the management of the child’s disease, and also determine the dosage properly. I learned many diabetes-related skills, and had confidence in the care of the child (A father)^(24)^.	Improve caring ability		
I started reading online about diabetes care. I obligated myself to take the responsibility of the care of my child myself (One mother)^(21)^.	Actively seeking out new information about the disease		
I read a book on healing. It helped me accept my child’s diabetes and also encouraged me to start taking care of my child (M10-mother of a 7-year-old girl)^(25)^.	Finding ways to relieve negative emotions		
If you let it, it (diabetes) will retreat into your life sooner or later, and we will always get back to normal (A father)^(24)^.	Encouraging children to live a normal life	Thinking positively about the future	
I told her to do whatever you want. You are just like everyone else (A mother)^(27)^.	Having confidence in having life back to normal		

## RESULTS

**Figure 1:**
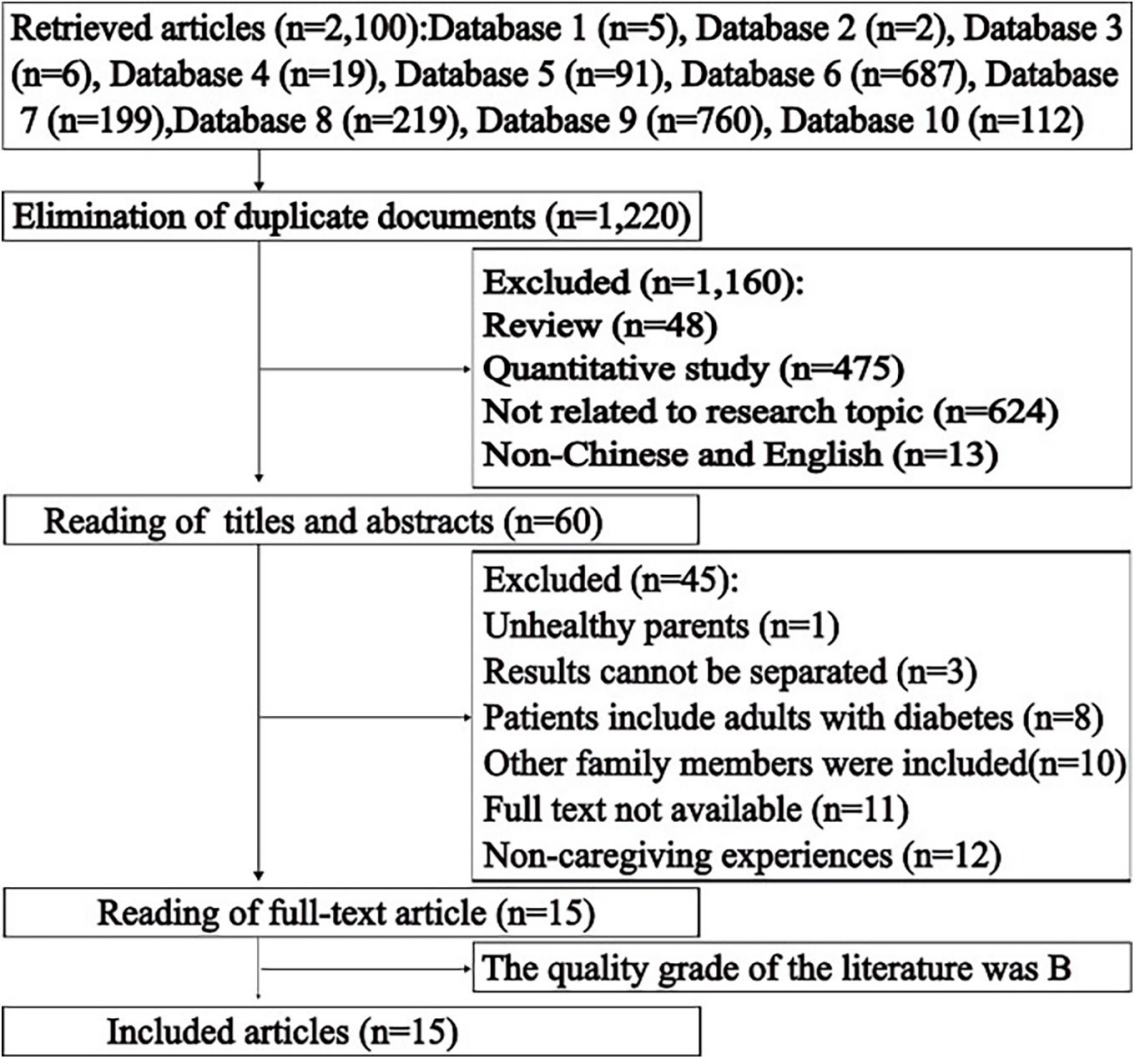
Flowchart of identification, selection and inclusion.

Base1: CNKI; Base2: VIP; Base3: CBM; Base4: Wang Fang; Base5: PubMed; Base6: Web of Science; Base7: CINAHL;

Base8: The Cochrane library; Base9: Scopus; Base10: Embase.

There were 2,100 articles (5 in CNKI, 2 in VIP, 6 in CBM, 19 in Wang Fang, 91 in PubMed, 687 in Web of Science, 199 in CINAHL, 219 in Cochrane Library, 760 in Scopus and 112 in Embase). Firstly, duplicate literature is searched and deleted by using the duplicate checking function of Note Express. A total of 880 duplicate articles were deleted, remaining 1,220 articles. After reading titles and abstracts, 1,160 studies were excluded. Of the 60 remaining articles, 45 were rejected because the parents of children in 1 study had physical or psychological problems; the results of 3 studies could not be separated; the patients of 8 studies included adults with diabetes; the subjects of 10 studies included other family members; the full text of 11 studies could not be obtained; and the assessment of noncaregiving experience was not available in 12 studies. Thus, out of 2,100 articles, 15 met the inclusion criteria and quality assessment. Therefore, the final sample consisted of 15 articles (Figure 1).

In the review, 15 articles show parents’ experience with the physical and psychological problems and challenges of caring for a child with T1DM. These studies were carried out in several countries on the five continents, demonstrating the global impact of caregiving. Participants included 162 mothers, 58 fathers and 13 parents (Chart 4). Through repeated literature reading, understanding and analysis, the results were summarized into 3 interrelated meta-topics.

**Chart 4:** The basic characteristics of included literature (n = 15).

Author	Country	Method of analysis	Participants	Context	Topic
Sullivan-Bolya et al.^(13)^	United States	Descriptive qualitative research	28 mothers	Home	Constant vigilance
Khandan et al.^(14)^	Iran	Phenomenon study	11 mothers	Home/workplace/hospital	Facing the care management challenges; Care in the shadow of concern; Hard life in the impasse of diabetes
Tong et al.^(15)^	China	Phenomenon study	12 mothers and 6 fathers	Hospital	Desire for information; Skill guidance needs; Seeking emotional support; Lack of social support
Iversen et al.^(16)^	Nordic countries	Phenomenon study	8 mothers and 7 fathers	Home/hospital/workplace/cafe	Striving to live an ordinary family life, yet feeling and living very differently
Rifshana et al.^(17)^	New Zealand	Phenomenon study	14 mothers and 3 fathers	Home/workplace	Problematic care for children with type 1 Diabetes Mellitus
Symons et al.^(18)^	New Zealand	Descriptive qualitative research	5 mothers and 4 fathers	Home	The destruction of family life; The impact on family relations; The influence of social psychology; Living with diabetes
Nurmi et al.^(19)^	Canada	Collective case study	13 parents	Failure to report	Hope the child can live like a normal person; Protecting children from harm
Smaldone et al.^(20)^	United States	Descriptive qualitative research	7 mothers and 7 fathers	Failure to report	Diagnostic experience: frustration, fear and doubt; Adjusting to diabetes; Negotiated development transformation
Moghadam et al.^(21)^	Iran	Descriptive qualitative research	20 mothers	Failure to report	Coping with the burden of care through personalized strategies and access to social support
Tong et al.^(22)^	China	Descriptive qualitative research	13 mothers and 7 fathers	Hospital	Continued psychological stress; Changes in family functions; Daily management challenges; Excessive financial burden; Lack of social support system
Rossiter et al.^(23)^	United Arab Emirates	Phenomenon study	4 mothers	Hospital	From disbelief and shock to acceptance of illness
Sullivan-Bolyai et al.^(24)^	United States	Phenomenon study	14 fathers	Home/hospital	From grief to action
Chan et al.^(25)^	Mauritius	Phenomenon study	11 mothers	Failure to report	Facing the disruption of life; Experiencing mixed emotions; Taking matters into one’s hands; Coping with life
Jinling Dai et al.^(26)^	China	Phenomenon study	10 mothers	Failure to report	Disease information needs; Individual health needs; Family support needs; Social support needs; Economic support needs
Haegele et al.^(27)^	United States	Phenomenon study	19 mothers and 10 fathers	Failure to report	The cost of type 1 Diabetes Mellitus; Supermom; Coping differently

### Meta-Topic 1: Parents Facing Multiple Physical, Mental and Life Challenges

#### Topic 1: At-Risk Physical Health

Parents of children with T1DM feel physically tired due to the heavy caring tasks. There are times when I’m completely drained. I sit down and cry. I ask God, “Why me?”. It’s challenging (M06-mother of a 5-year-old girl)^(25)^. Because the child’s parents also need to continuously measure blood sugar at night, resulting in reduced sleep and poor sleep quality, gradually depriving parents of sleep. I have become an insomniac. I don’t sleep at night as every 2-3 hours I have to check on him (…) I sleep only 2-3 hours. It’s my son’s diabetes that has made me like that (M07-mother of a 9-year-old boy)^(25)^.

#### Topic 2: Complex Emotional Experience

When a child is just diagnosed with T1DM, parents find it difficult to accept the fact that their child is ill, often choosing to deny and escape. I could not believe that my child had diabetes. I took him to different doctors (One mother)^(21)^. Fearing of managing their child’s illness, especially when it comes to insulin injections. It was like you were killing your own daughter… I don’t think that I’m going to be able to do it (One mother)^(20)^. A father described learning to manage his child’s diabetes in the hospital as a “kind of baptism by fire”^(20)^. Also, parents tend to blame themselves for their child’s illness and feel guilty about their child’s illness. I feel guilty that I may have done something wrong that has resulted in my child’s diabetes as I’m the one who feeds him and takes care of him (M07-mother of a 9-year-old boy)^(25)^. At the same time, he felt sadness and pity for his child’s illness. I went through a grieving process, not grief for dying... it’s about the loss of your child’s health (One mother)^(18)^.

Furthermore, parents also suffer from misconceptions about T1DM. Society’s perception of diabetes is backward and even wrong. Her peers were afraid to drink from her glass as they thought that it was contagious...(M10-mother of a 10-year-old girl)^(14)^. They experienced shame related to T1DM, fearing that the child’s future will be affected by T1DM. I was so worried about the consequences of spreading the news among them, which may have a negative or bad effect on my daughter’s future (FATIMA)^(23)^. Sometimes, to ensure a child’s safety, parents need to maintain a constant state of high stress. Diabetes is an underrated condition: you look healthy—nothing is visible. To be parents in this situation implies that you live in a state of perpetual attention (Mother child 7)^(16)^. In addition, parents of sick children also have an ambivalence, i.e., they want to reduce their own care burden but cannot let go of the care responsibility to others. There are family and friends ready when we need time for ourselves. But it is difficult to let go, and we think about all the considerations we must make all the time. It is difficult for us to leave this responsibility to others (Mother child 7)^(16)^. At the same time, parents are also worried about the child’s future, including worries about the child’s future body and life. I am always thinking about his future. I wonder what will happen to his body. Can he be successful in his life? I do not know; the future is unclear (M:3)^(14)^. She worries about her marital status. It’s so hard for a girl, especially in our society. When she wants to get married, will anyone be interested in her? These issues worry me about my child’s future (M:4)^(14)^.

#### Topic 3: Taking Care of Lack of Knowledge and Ability

Parents often lack knowledge related to the disease. We are confused about why blood sugar control is not very good, I don’t know what to do, I want to know all about blood sugar control (One mother)^(26)^. At the same time, parental care skills need to be improved. I was confused at the beginning and did not know where to start. I had never practiced insulin injection before. I still remember how my hands trembled when I injected my child the first time, causing me to inject too much insulin (DM17)^(15)^.

#### Topic 4: Limited Personal Development and Social Activities

Parents may choose to change their career plans because they need more time to take care of their children. Now I rarely focus my energy on the company [I work for]; just get off work as soon as possible. I used to leave early and return late to make money. Now I quit my job and found a new one that can make a living while taking care of my child (DM5)^(21)^. Since caring for a child takes up almost all of the free time, parents’ normal social interaction is bound to be affected. Going to family gatherings has become very difficult, because we have to watch his diet (One mother)^(21)^. Since my child had a ketosis coma, I have never dared to relax again. I have lost myself. Every day in the year, there are no more visits to relatives or friends (DM10)^(22)^. At the same time, parents have reduced personal time flexibility, losing spontaneity and flexibility in daily life. I revolve around my children almost all day, and I don’t have any time to myself (One mother)^(18)^.

#### Topic 5: The Reconstruction of Family Dynamics

When there is a sick child in the family, parents tend to devote more energy to the sick child and inevitably pay less attention to the other children, which makes the other siblings feel neglected and have bad feelings. I spend a lot of time with Ahmed. This has made his siblings dissatisfied (One mother)^(21)^. In addition to the impact on siblings, it also involves the division of responsibilities between spouses in the family. We ordered beepers and we would beep. Every time… (she) did a (blood glucose) test we would beep the number… she’d just beep the number and I would call her and we would consult each other (A father of a 4-year-old child)^(20)^, and support each other. I was…completely overwhelmed, but my husband… we were a team, so that was huge… we did everything together. We did the checking her together and we… were figuring it out together so that really helped a lot because we didn’t feel like we were completely alone (One mother of a 11-month-old daughter)^(20)^. However, the heavy burden of care can also make it difficult for parents to balance caring for a child and managing the family’s daily life. I have too many chores and things to take care of besides taking care of him, but it is difficult for me to juggle them (M07-mother of a 9-year-old boy)^(25)^. Also, diabetes-related family conflict is inevitable. What shall I say…near fights where we have accused each other like. Why did you forget this? Don’t you know that this food contains lots of carbohydrates? Yes, we have had a few of those (Father child 8)^(16)^.

#### Topic 6: Heavy Economic Burden

The long-term treatment of children with T1DM is expensive, and parents are under greater financial pressure. The test strip and the insulin needle are too expensive. We did not have such costs earlier, but now we have to save by cutting down on family expenses for the sake of our child (M:7)^(14)^.

### Meta-Topic 2: Parents’ Lack of a Full Range of Outside Support

#### Topic 7: Insufficient Family Support

In the process of caring for children with T1DM, the father takes less responsibility than the mother, which is reflected in the absence of the father’s caring role.… a lot of things I don’t think he (father) does right with this, and I’m sure he thinks I micro-organize the whole thing… he doesn’t get the calls from the nurse. He’s not watching the meter all day with every number that’s coming up, and so I think it’s a little more stress free for him (One mother of a 9-year-old girl )^(20)^. Sometimes, parents also suffer from the lack of understanding of relatives. My son is a little uncomfortable; they (family) are putting the responsibility on me, the child does not cooperate with me, I can do nothing, they should understand my difficulties more (One mother)^(26)^. However, I still hope that my family members can participate in the care work together. I hope the whole family can help me share a little, because my ability is limited, and if my family members can help take care of me, I can feel more relaxed and the care effect will be better (One mother)^(26)^.

#### Topic 8: Insufficient Professional Support

Parents of children with T1DM crave professional guidance from healthcare professionals. I really want the doctors and nurses to tell me what kind of food that my child can and cannot eat, in what amount, and how to prepare it, so I can write them all down in a little notebook. I have a hard time figuring out the calories of the diet. I wish I could ask the doctors and nurses to prepare a list of recipes so I can follow them; otherwise, it is too difficult for me to prepare the meals for my child (DM7)^(15)^. On the other hand, parents have difficulty accessing authoritative information. Nowadays, we can find all kinds of information on the Internet, but there are always examples of falsehoods on the Internet. The more the information that I browse, the more I want to know, and I feel panicky deep down. I particularly hope that there will be a professional platform where I can ask question when I encounter problems that I cannot handle and receive timely answers from doctors and nurses (DM8)^(15)^.

#### Topic 9: Insufficient Social Support

Parents of T1DM children expect better social security to share some of the family pressure. The children do not have medical insurance; these test strips, insulin cannot be reimbursed. It would be nice if some drugs were covered by Medicare, as they are for type 2 diabetes (One mother)^(26)^. At the same time, most children with T1DM are at school age and have been in school for a long time, but school configuration is not perfect, causing parents to worry about their children’s school situation. Since my child has started going to school, my heart really trembles as soon as the school calls, and I am afraid that my child will have an accident. Teachers at school do not know how to take care of diabetic children, and the schools generally have no nurse specialist. I am really afraid that there will be an accident (DM13)^(15)^.

#### Topic 10: Unreasonable External Expectations

Some parents felt that the hard work and effort they put into the care process was taken for granted by the family and the healthcare staff. The family will tell me that you are the mother of the child and you must take care of him (One mother)^(17)^, and the healthcare staff had unreasonable expectations about what could be achieved. I feel anxious every time I go to the clinic. They (healthcare staff) don’t understand the reality of living with a T1DM child, that I’ve done my best and still don’t meet the requirements, and he feels that I should be able to take care of the child (One mother)^(20)^. This brings a huge psychological gap to children’s parents.

### Meta-Topic 3: Parents’ Caregiving Role Competency Enhanced to Adjust to the New Life

#### Topic 11: Self-Empowerment, Actively Responding to Life Changes

Over time, parents gradually accept that their child has diabetes. But over the time, the situation has been changed and become almost ordinary and acceptable to us (KHULUD). Gradually over time, we adapted to the new situation (BASMAH)^(23)^, and also gradually adapt to the role of caregiver. Taking care of my daughter, it’s not that I think I can or can’t, but that I must be able to do. It’s like a parent’s obligation. If I can’t, what else can she do? It’s not something I can or can’t do. It’s something I must be able to do (DM6)^(22)^. The long-term caregiving experience also improved parents’ caregiving ability and confidence. As time went by, I felt that I could handle the problems in the management of the child’s disease, and also determine the dosage properly. I learned many diabetes-related skills, and had confidence in the care of the child (A father)^(24)^. Parents also actively seek out new information about the disease and take responsibility for care. I started reading online about diabetes care. I obligated myself to take the responsibility of the care of my child myself (One mother)^(21)^. At the same time, parents seek ways to relieve their own negative emotions, reduce emotional stress and better care for the child. I read a book on healing. It helped me accept my child’s diabetes and also encouraged me to start taking care of my child (M10-mother of a 7-year-old girl)^(25)^.

#### Topic 12: Thinking Positively About the Future

Parents gradually adjust to their child having diabetes and begin to look at life with optimism, not only confident in returning to normalcy. If you let it, it (diabetes) will retreat into your life sooner or later, and we will always get back to normal (A father)^(24)^, also encouraging the child to live as a normal person (I told her to do whatever you want. You are just like everyone else (A mother)^(27)^). My child is fine, he is a normal child, he just needs insulin, that’s all (One mother)^(19)^.

## DISCUSSION

The findings of this study reveal that parents experience a spectrum of negative emotional states, including worry, fear, sadness, compassion, and a pervasive sense of vigilance throughout the caregiving process. This emotional burden remains substantial over time and aligns with the findings of Saßmann et al.^(28)^. If not addressed, these persistent negative emotions can detrimentally affect not only parents’ quality of life but also the well-being and diabetes management efficacy for children with T1DM. It is, therefore, critical to acknowledge the mental health challenges faced by these parents and to provide them with professional support and guidance.

In response to these challenges, healthcare professionals could establish group training sessions designed to enhance caregiving skills, offer emotional support, and facilitate confidential interactions among families. Such settings allow for peer learning in a supportive atmosphere, where role modeling and shared problem-solving are encouraged^(28)^. Moreover, for parents constrained by limited time flexibility, web-based mindfulness interventions offer an alternative pathway to gaining autonomy^(29)^. Through mobile applications or online platforms, parents can access mindfulness training and skill-building resources, enabling them to adopt new perspectives on caregiving challenges and to employ effective stress management techniques. Enhancing their psychological well-being can, in turn, encourage the adoption of positive parenting strategies and improve the quality of care provided to children. Additionally, the study highlights the ambivalence felt by parents desiring to alleviate their caregiving burden without compromising their responsibility. This concern often stems from doubts regarding secondary caregivers’ caregiving competencies.

To mitigate these concerns, it is essential for professional medical teams to develop structured educational programs and practical training platforms^(5)^. These initiatives should aim to equip secondary caregivers with essential T1DM knowledge and insulin administration skills, thereby bolstering parents’ confidence and trust in their caregiving capabilities.

T1DM, recognized as a familial condition, exerts a profound impact on both family members and familial dynamics, potentially disrupting the balance within the family structure. This observation aligns with the findings presented by Chan et al.^(30)^. The persistence of imbalances within familial relationships may escalate the risk of familial conflicts, amplify the caregiving burden on parents, and negatively influence T1DM management in affected children. Consequently, it is advisable for healthcare professionals to adopt a holistic approach to supportive interventions that encompasses the entire family. This involves acknowledging the roles played by siblings and the division and support of spousal responsibilities, crafting and executing comprehensive family-centric support initiatives, and fostering a balance in family dynamics.

Healthcare providers are positioned to offer family empowerment strategies^(31)^, enabling family members to collaboratively devise management and caregiving strategies for children diagnosed with T1DM. Interactions among siblings, often marked by mutual support, teaching, comforting, and caregiving^(32)^, suggest that their increased involvement in the care of children with T1DM could alleviate caregiving burden on parents and cultivate a deeper sense of empathy towards their afflicted siblings, thereby enhancing familial bonds. Furthermore, a clear delineation of responsibilities within the family can significantly contribute to disease management and the distribution of caregiving pressures^(33)^. Therefore, healthcare professionals should assist parents in forming a caregiving partnership, encouraging a more collaborative effort in managing both direct and indirect caregiving responsibilities for children with T1DM. This approach aims to enhance the efficacy of family-based care, mitigate familial conflicts, and strengthen family unity.

Furthermore, misconceptions regarding the care of children with T1DM contribute to the formation of unrealistic expectations and perceptions among family members and healthcare professionals. This observation aligns with the findings of Kimbell et al.^(34)^. Such unfounded expectations and perceptions can lead to a significant psychological disconnect for parents, impeding their development as caregivers and adding undue psychological stress and burden. Consequently, rectifying these misconceptions and bolstering parents’ role identity and perceived benefits of care is crucial.

Healthcare professionals must grasp the complexities and dynamic nature of diabetes management within the family context. They should establish realistic goals and expectations tailored to each family’s unique circumstances, address children’s needs, and remain cognizant of the stress and challenges parents face in managing diabetes amidst the broader scope of family life^(35)^. Furthermore, it is essential for medical staff to acknowledge the sacrifices parents make in caring for children with T1DM, offering acknowledgement and praise. This acknowledgment can guide parents towards appreciating the positive feedback from the caregiving process, thereby enhancing their role identity and perceived benefits from providing care.

Furthermore, healthcare professionals ought to incorporate empathy into their interactions with parents of ill children. The integration of empathetic communication not only enhances the quality of interactions between nurses and patients but also strengthens the trust between them^(36)^, which in turn facilitates a deeper understanding by parents regarding their children’s conditions and helps establish realistic expectations. Consequently, healthcare providers should maintain active listening, acknowledge parents’ efforts, and proactively demonstrate a readiness to assist, thereby fostering empathy in nurse-patient interactions and paving the way for a more profound comprehension of parents’ caregiving experiences and the rectification of any unwarranted external expectations.

Moreover, upon their children’s diagnosis, parents are thrust into their caregiving role, often leading to an initial reliance on negative coping mechanisms such as denial and avoidance. This transition to positive coping strategies, characterized by acceptance and coping, may be protracted, aligning with the findings of Lowes et al.^(10)^. The persistence of a negative coping approach not only exacerbates caregiving stress but also impedes the transition to and acceptance of the caregiving role. As such, it is imperative for healthcare providers to intervene promptly upon identifying negative coping behaviors in parents, guiding them towards more constructive coping mechanisms to hasten the process of adaptive coping.

The manner in which caregivers respond is intricately linked to their intrinsic motivation for embracing the caregiving role^(37)^. Refining and directing this motivation towards caregiving can significantly enhance both the caregiving experience and its outcomes. Thus, healthcare professionals are encouraged to mentor caregivers, particularly parents, to cultivate a caregiving ethos rooted in love. This approach not only posits caregiving as an extension of emotional commitment but also facilitates caregivers in finding deeper meaning and growth within their roles. By shifting caregivers’ motivations from purely altruistic to include elements of self-benefit and by nurturing a positive response through the exploration of personal internal motivations, a transformative caregiving dynamic can be established.

Drawing on Parkes’ Social Psychological Transformation Theory^(38)^, individuals facing substantial life alterations—from avoidance to coping—require robust emotional support and the discovery of new worldviews to effectively navigate these changes. Consequently, healthcare professionals must be attuned to and address the negative coping mechanisms of caregivers, fostering open discussions about their emotional experiences and providing the necessary emotional support. By assisting caregivers in integrating the changes that diagnosis brings, encouraging the establishment of new life patterns, and supporting adjustment and adaptation, healthcare professionals can significantly diminish the duration required for adaptive coping.

This study, however, has its limitations. It is restricted to literature in Chinese and English, potentially overlooking vital information available in other languages. Furthermore, our research identifies a significant gap in exploring the distinct caregiving burdens shouldered by fathers and mothers. This disparity is critical to address, given that mothers are often the primary caregivers and take greater responsibility in care management than fathers. Future research should aim to understand the unique experiences and burdens of both mothers and fathers in their caregiving roles. This insight will allow healthcare professionals to identify stressors and provide more targeted support.

## CONCLUSION

In this study, we conducted a qualitative meta-synthesis of parents’ caregiving experiences with children diagnosed with T1DM. Our findings reveal that these parents encounter a spectrum of physical, psychological, and lifestyle challenges in their caregiving journey. There is a noticeable lack of comprehensive external support for these parents, yet their caregiving competencies tend to improve as they adapt to their new roles. We recommend that healthcare professionals implement strategies such as group training and online mindfulness interventions to bolster these parents’ mental well-being. Additionally, developing tailored and inclusive family support plans is crucial to promote a dynamic balance within families. It is equally important to enhance medical staff empathy, establish realistic family management goals, and improve parents’ sense of accomplishment in caregiving. Moreover, adjusting the motivations behind parental caregiving and enhancing the overall care experience and outcomes should be a focus.
